# The Design of a Reflection Electron Energy Loss Spectrometer Attachment for Low Voltage Scanning Electron Microscopy

**DOI:** 10.3390/ma14247511

**Published:** 2021-12-07

**Authors:** Jonathan Chuah, Anjam Khursheed

**Affiliations:** Department of Electrical and Computer Engineering, National University of Singapore, Singapore 117583, Singapore; jchuahwj@nus.edu.sg

**Keywords:** scanning electron microscopy, electron energy spectroscopy, energy analyzer, energy resolution

## Abstract

This paper presents the design of a reflection electron energy spectrometer (REELS) attachment for low voltage scanning electron microscopy (LVSEM) applications. The design is made by carrying out a scattered electron trajectory ray paths simulation. The spectrometer attachment is small enough to fit on the specimen stage of an SEM, and aims to acquire nanoscale spatially resolved REELS information. It uses a retarding field electrostatic toroidal sector energy analyzer design, which is able to lower the kinetic energies of elastically backscattered electrons to pass energies of 10 eV or less. For the capture of 1 keV BSEs emitted in the polar angular range between 40 to 50°, direct ray-tracing simulations predict that the spectrometer attachment will have an energy resolution of around 0.4 eV at a pass energy of 10 eV, and 0.2 eV at a pass energy of 5 eV. This predicted performance will make it a suitable REELS attachment for SEMs that use field emission electron sources.

## 1. Introduction

There is at present a critical need to develop material science analytical tools for low voltage scanning electron microscopes (LVSEMs). LVSEMs have many well-known advantages over conventional scanning electron microscopes (SEMs). They have higher signal yields, a smaller beam/specimen interaction volume, greater surface information, and the possibility of minimizing charging effects while inspecting non-conductive specimens [1]. LVSEMs have now largely replaced the use of conventional SEMs for high-resolution imaging applications and greatly extended the kinds of specimens that can be observed [2,3,4,5]. However, they still have the disadvantage of not being able to function with the energy dispersive X-ray (EDX) spectroscopy technique. EDX only functions well for primary beam landing energies of 6 keV or higher. The landing energies inside LVSEM systems, of 2 keV or less, are not high enough to generate the necessary atomic transitions that emit characteristic X-rays [6,7]. The difficulty of using EDX with LVSEMs means that a sample after LVSEM inspection often needs to be reexamined in a conventional SEM at a lower image resolution with an EDX attachment.

One possible way forward is to develop electron energy spectroscopy attachments for LVSEMs and use methods such as reflection electron energy loss spectroscopy (REELS). REELS involves acquiring the energy spectrum of elastically scattered backscattered electrons (BSEs), particularly those that have loss energies up to hundreds of eV [8]. REELS can measure semiconductor band gaps [9] and provide chemical analysis information [10]. A closely related technique also relying on the acquisition of elastic backscattered electron energy spectra is elastic peak electron spectroscopy (EPES). EPES has been proposed for SEMs to improve image contrast and acquire inelastic mean free path information [11].

The research work presented here follows from the recent success of using secondary electron energy spectroscopy (SEES) inside an SEM for various different material analysis applications. SEES was successively used to acquire bulk valence band density of states information [12], characterize semiconductor wafers and measure dopant concentration distributions [13], and track the build-up of charge in samples having insulator layers [14,15]. These achievements were made possible by using a compact high signal-to-noise secondary electron (SE) toroidal electrostatic sector energy analyzer attachment, small enough to fit on to an SEM’s specimen stage [16,17,18].

The research work presented here carries out a direct ray-tracing simulation to redesign the present SE toroidal energy analyzer attachment to acquire REELS information inside the SEM. The REELS application inside an SEM requires that the analyzer attachment energy resolution be smaller than the energy spread of the primary electron beam. Therefore, the energy resolution needs to be below 0.5 eV for SEMs that use a thermal field emission Schottky source and be better than 0.3 eV for SEMs that use a cold field emission source [19]. Assuming that the SEM will be operated in a low voltage mode of operation, where the primary beam landing energy is typically 1 keV or below, the relative energy resolution of the REELS toroidal energy analyzer attachment, therefore, needs to be better than 0.05%. In order to achieve this, a retarding field version of the present toroidal energy analyzer SEM attachment was designed, capable of slowing down the kinetic energies of BSEs to pass energies of 10 eV or less at the same time, maintaining its high performance second-order focusing optical characteristics.

## 2. Materials and Methods

The research work presented here redesigns the SE toroidal energy analyzer attachment for the purpose of acquiring REELS information inside the SEM. This section first describes the original analyzer design in detail, presents the modifications made for the new retarding field analyzer design, and discusses the computational methods used to simulate and optimize the design’s performance.

### 2.1. The Previous SE Toroidal Energy Analyzer Attachment

**Figure 1:**
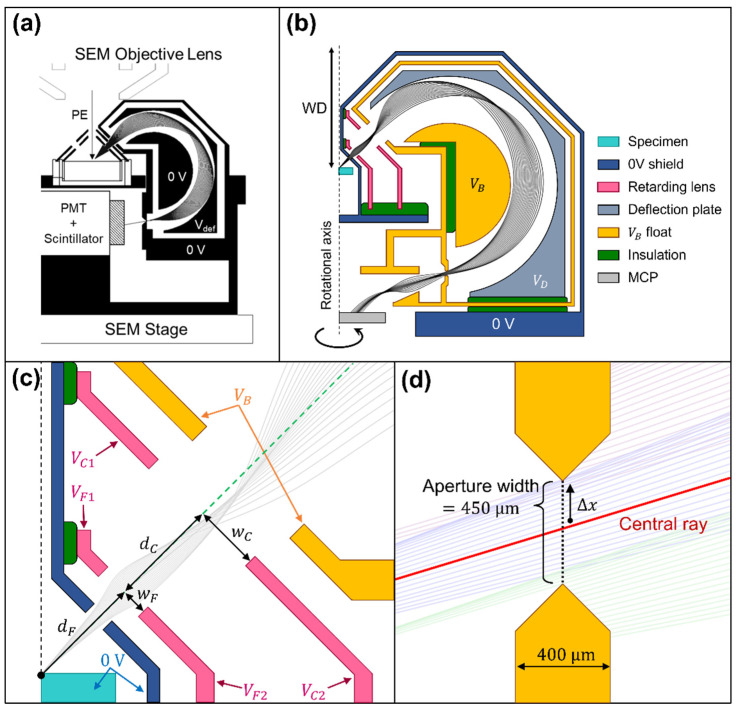
Schematic drawing of SEM toroidal energy spectrometer attachment designs. (**a**) A previous design for secondary electron energy spectroscopy (SEES). (**b**) Present design for reflection electron energy spectroscopy (REELS). (**c**) Retarding column lens section in the REELS analyzer design. The four electrodes located between the outer 0 V casing plate and the analyzer entrance plate held at voltage $V_{B}$ control the way the incoming electrons are slowed down before they enter the analyzer, and ensure that the second-order focusing properties of the analyzer design are preserved. (**d**) Exit aperture in the REELS analyzer design.

Figure 1a shows the previous secondary electron toroidal energy analyzer attachment design schematic drawing. The attachment is mounted onto the specimen stage inside the SEM specimen chamber, and is aligned to the primary beam axis in situ using the SEM secondary electron (SE) detector image. The height of the stage is adjusted to give the minimum operational working distance of 15 mm. An analyzer entrance aperture limits the entrance polar angular range to be 45° ± 8°, and the effective azimuthal angular collection range used is 100°. All other electrodes in Figure 1a are held at ground potential, except the outer deflector plate voltage analyzer, $V_{def}$, and the scintillator on the front of the photomultiplier (PMT) detector. $V_{def}$ is ramped through a series of negative voltages in the 0 to −25 V range, and the voltage on the scintillator is set to between 3 to 5 kV. The analyzer is of the band-pass type, where the entrance/exit apertures of the analyzer allow for the detection of a transmitted bandwidth $\Delta E_{p}$, centered around a pass energy of $E_{p}$. Varying the analyzer deflection voltage $V_{def}$ varies the pass energy $E_{p}$, effectively sampling through the SE energy distribution with an energy width of $\Delta E_{p}$_._ The pass energy and outer deflection plate voltage relationship are given by, $E_{p}=1.67\cdot \left|V_{def}\right|$. Since high energy resolution is not required for the SEES application, the analyzer is deliberately run in a limited aperture mode, where a relatively large exit aperture is used (up to a width of 0.5 mm), giving an overall relative energy resolution of 2 to 5%. A prominent exit aperture helps maximize the analyzer’s output signal-to-noise ratio, required for most SEES applications.

### 2.2. The Retarding Field Toroidal Analyzer Attachment Design

Figure 1b shows the schematic drawing of the redesigned REELS toroidal energy analyzer attachment. The overall geometry and dimensions of the analyzer attachment are unchanged. The main difference lies in inserting an extra float electrode held at the potential of $V_{B}$, and a retarding lens column located in front of the analyzer entrance. The purpose of the float electrode is to provide a new reference potential for the interior part of the analyzer. Instead of the outer deflection plate potential varying with respect to a grounded inner deflection plate, as in the original analyzer design (Figure 1a), it is now ramped with reference to an internal deflection plate voltage of $V_{B}$. The voltage $V_{B}$ on the float electrode and internal deflector plate is used to control the analyzer’s pass energy. For instance, incoming elastic 1 keV BSEs with a $V_{B}$ set to −990 V will be retarded to have an analyzer pass energy ($E_{p}$) of 10 eV, instead of 1 keV. The outer deflection plate is then ramped through a series of negative voltages with respect to $V_{B}$, producing a transmitted pass energy bandwidth, as in the original analyzer design, but the main difference now is that the amount of energy dispersion on the exit aperture plane is produced with respect to the new retarded pass energy of 10 eV instead of 1 keV, and is therefore increased by two orders of magnitude.

After going through the exit aperture, electrons are accelerated down onto a microchannel plate (MCP) detector held at 0 V. The MCP detector here replaces the previous scintillator/PMT combination as the new analyzer attachment is to provide for the possibility of two sector analyzers operating in parallel, one for SEES, and one for REELS. If only the REELS mode is used, the MCP entrance grid need not be biased (0 V), since elastically scattered BSEs already have enough energy to be detected. If only the SEES mode is used, the entrance grid of the MCP need only be biased to around 100 V for optimal detection.

Figure 1c shows a schematic drawing of the retarding lens column. There are four electrodes located between the analyzer’s outer 0 V casing plate and the analyzer entrance plate (at voltage $V_{B}$). The electrode voltages, $V_{F1},V_{F2},V_{C1},V_{C2}$*,* and the distances, $d_{F},d_{C},w_{F},w_{C}$, were treated as free parameters in an optimization procedure based upon maximizing the transmitted polar angular spread while at the same time keeping the relative energy resolution to be better than 0.05%. To make room for the electrodes in the retarding lens column unit, the diameter of primary beam passing hole is now reduced to be 0.5 mm, compared to 2 mm in the original analyzer attachment design.

Figure 1d shows a schematic drawing of the region around the analyzer exit plane. The output beam spot size is quantified by the transverse distance Δx on the exit aperture plane and is a function of both the entrance polar angular spread, Δθ, and electron energy dispersion. The dependence with Δθ comes from geometric aberration, which on the Gaussian focusing plane of the original toroidal analyzer design, takes a cubic form ($\Delta \theta^{3}$), characteristic of a second-order focusing action [16]. The energy dispersion term, like most electric sector analyzers, varies linearly with energy dispersion ΔE and is usually expressed in terms of the energy dispersion parameter, D, where Δx=DΔE. The energy spread reaching the electron detector is therefore determined by the exit aperture width w, and is given by ΔE=w/D. The latter term is usually referred to as an aperture limited energy resolution, $\Delta E_{DIS}$. An estimate of the aberration limited energy resolution, $\Delta E_{AB}$, can then be found from the RMS value of the geometric aberration spot size, $\Delta x_{RMS}$, $\Delta E_{AB}=2\Delta x_{RMS}/D$. The total energy resolution ($\Delta E_{tot}$) can be estimated by adding the energy dispersion ($\Delta E_{DIS}$) and the geometric RMS spot ($\Delta E_{AB}$) terms in quadrature,

(1) $${\left(\Delta E_{tot}\right)}^{2}={\left(\Delta E_{AB}\right)}^{2}+{\left(\Delta E_{DIS}\right)}^{2}$$

The exit aperture width in the retarding field toroidal analyzer design was fixed to w= 0.45 mm, a value well suited for the SEES application.

Although the new toroidal energy analyzer attachment was designed primarily with the REELS application in mind, steps were taken to ensure that it could also be used for SEES simply by applying different voltages to its electrodes. This feature gives it the capability of performing REELS and SEES in a straightforward sequential way, enabling it to acquire more complete material information about the sample under examination.

### 2.3. The Simulation Procedure

The performance of the toroidal energy analyzer attachment was computed using a direct ray-tracing simulation approach, where the trajectory paths of scattered electrons leaving the specimen at different energies and angles were traced through rotationally symmetric electrostatic field distributions. The commercial software Lorentz 2EM v9.3 by Integrated Engineering Software was used to define the analyzer’s electrode layout, solve for the electric field distribution, and compute the resultant electron trajectories [20]. Lorentz 2EM is able to perform accurate charged particle ray-tracing using a suite of high-performance PDE and ODE solvers. Accurate field solutions are computed efficiently via the semi-analytical boundary element method (BEM) in conjunction with adaptive meshing algorithms, which adapt the number of charge segments on electrode boundaries in accordance with the local electric field strength. Then, to compute the electron trajectories, a variable step-size fifth-order Runge–Kutta solver is available, which varies the step-size according to truncation error. Many steps are automatically selected in regions of high electric field strength, while large step-sizes are used in regions of low electric field strength. To ensure that the simulation accuracy was sufficient, the electric field distribution and electron trajectory paths were re-computed with error tolerances in the BEM and ODE solvers that were successively halved, until the final simulated parameters such as beam spot size did not change by more than 1%.

To obtain sufficient resolution and transmittance for the REELS application, the design of the toroidal analyzer was posed and solved as a constrained optimization problem. The optimization procedure started with first setting error tolerances for each parameter and monitoring the spot size on the exit aperture plane for a range of different input polar angles and energies. The analyzer design was optimized for 1 keV elastically scattered BSEs being retarded down to a pass energy of 10 eV. Then, a nested procedure was used to perform the optimization: an inner loop optimized the focusing for a given entrance polar angular range, while an outer loop increased the polar angular range in steps until a maximum range was attained.

For the inner loop, a pass energy was set using the toroidal sector voltages $V_{B}$ and $V_{D}$, and a fixed entrance angular range, θ∈[π/4−Δθ,π/4+Δθ], was chosen. Then, the retarding lens voltages, $\left[V_{F1},V_{F2},V_{C1},V_{C2}\right]$, and geometric parameters, $\left[d_{F},w_{F},d_{C},w_{C}\right]$, were chosen as optimization variables, while the objective function to be minimized was chosen to be the rays’ RMS spot size, $\Delta x_{RMS}$, evaluated at the exit aperture plane. This was also done for different exit aperture positions, in order to determine the optimal position of the exit aperture plane along the ray path. Additionally, to avoid unrealizable lens configurations such as small inter-electrode distances and self-intersecting geometries, linear constraints were imposed on the four geometric parameters. The resulting constrained optimization problem was solved with MATLAB’s lsqnonlin solver using the reflective trust-region algorithm [21]; the forward simulation passes were performed by Lorentz 2EM, and derivative information was computed by the MATLAB solver using finite differences. The procedure was also re-run multiple times with different initial parameter values to verify that the final computed solution was globally optimal.

The result of each optimization run was a spectrometer configuration that, for the specified entrance angular range, focused the beam into the smallest achievable spot size at the exit aperture plane, from which an energy resolution estimate was made. In order to obtain the largest possible angular range, the optimization procedure was repeated for different angular ranges, increasing Δθ in steps of 0.5°, until the energy resolution corresponding to the spot size reached the target 0.05% value.

## 3. Results and Discussion

### 3.1. Toroidal Analyzer Simulated Performance in REELS Mode

**Figure 2:**
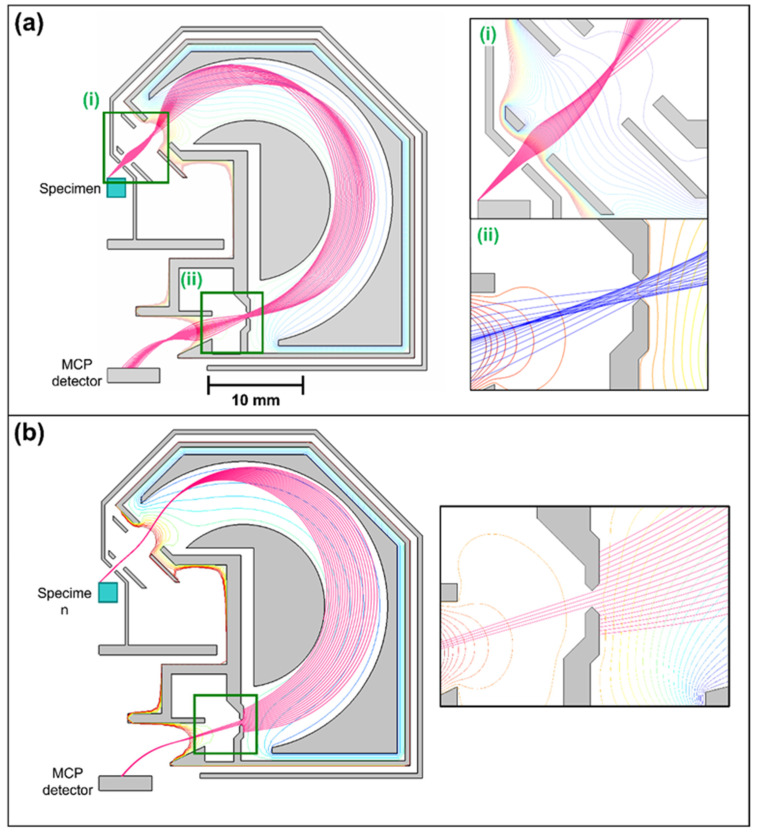
Direct ray tracing of BSEs in the optimized toroidal energy analyzer attachment design for a pass energy of 10 eV. (**a**) 1 keV BSEs emitted over a 40° to 50° polar angular range (i) Around the entrance region (ii) Around the exit region. (**b**) BSEs emitted in the 999 eV to 1001 eV energy range in 0.1 eV steps with a polar emission angle of 45°.

Figure 2 shows trajectory paths of BSEs in the optimized analyzer design for a central BSE emitted energy of 1 keV and pass energy of 10 eV. There is an intermediate focal point between the deflection plates (Figure 2a), similar to the one inside the original analyzer design (Figure 1a). Electrons are brought to a sharp focus before entering the analyzer (Figure 2(ai)), and this causes the exit focal point to move beyond the aperture plane (Figure 2(aii)). At first glance, this does not appear to be an optimal solution since the exit focal position is pushed out far beyond the exit aperture plane position. However, in the region beyond the exit aperture, the wider angles strongly diverge (Figure 2(aii)), which has the effect of increasing the overall RMS spot size. In addition to this, the energy rays appear to be converging as they pass through the exit aperture (Figure 2b), indicating that at the point of focus for the smaller angles (Gaussian plane), there is less energy dispersion. These two effects combine to make the simulated aberration limited energy resolution ($\Delta E_{AB}$) on the exit aperture plane to be smaller than its simulated value on the Gaussian plane.

**Figure 3:**
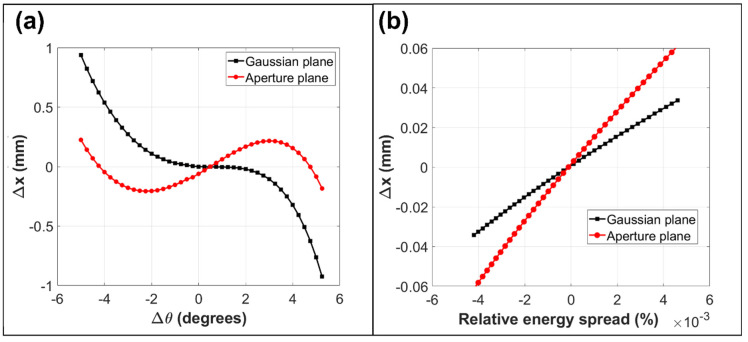
Simulated focusing action at the exit aperture and Gaussian planes for 1 keV BSEs retarded down to a pass energy of 10 eV. Exit distance (Δx) from the central ray as a function of (**a**) emission polar angular spread, and (**b**) the relative energy spread.

Figure 3 shows the simulated focusing optics on the exit aperture plane for the optimized retarding field analyzer design. In Figure 3a, the spot size is plot as a function of input polar angular spread and is compared to its predicted variation on the Gaussian plane. The Gaussian plane was obtained by projecting the small angle rays from the exit aperture plane, and was found to lie 3.77 mm beyond it. The actual simulated point of focus for the small-angle rays is not representative of the analyzer exit optics since it does not lie in a field-free region. The fact that the Gaussian plane variation takes a regular cubic form, confirms that the desirable second-order focusing characteristics of the original toroidal analyzer have been preserved in the optimized retarding field analyzer design. The main difference is in the location of the Gaussian plane, which appears to be pushed out several millimeters beyond the original point of focus. Figure 3b indicates that the simulated energy dispersion is smaller at the Gaussian plane than at the exit aperture plane, confirming that the energy rays converge as they exit the analyzer (Figure 2b). From an energy dispersion point of view, the exit aperture plane should therefore be placed as close as possible to the toroidal deflection plates.

The simulation results shown in Figure 3 indicate that the spot size due to both angular and energy dispersion are predicted to be better on the exit aperture plane than on the Gaussian plane. These results are consistent with the ray-tracing information presented in Figure 2. The overall spot size is smaller on the exit aperture plane because the wider-angle rays form a point of focus there (Figure (2aii)), while the energy dispersion is lower at the Gaussian plane because the energy rays are converging as they exit the analyzer (Figure 2b).

Table 1 shows the analyzer electrode voltage values and focusing parameters in the final retarding field toroidal analyzer design for 1 keV BSEs being retarded down to the pass energies of 10 eV and 5 eV. A similar optimization procedure was used to derive the electrode voltage values for the 5 eV pass energy case. The RMS simulated spot size on the exit aperture plane was minimized while maximizing the transmitted polar angular spread. This resulted in the 5 eV pass energy simulated exit RMS spot size being similar to the one that was obtained for the 10 eV pass energy case. The simulated aberration limited energy resolution at the exit aperture plane ($\Delta E_{AB}$) is a factor of two better than its value on the Gaussian plane, for both 10 eV and 5 eV pass energies. The overall relative energy resolution simulated for the 5 eV pass energy, of 0.0199%, is approximately half its 10 eV pass energy value, of 0.0395%, while the predicted reduction in transmittance in going to the lower pass energy is only 6% (reduction of the polar angular spread from ±5° to ±4.7°). This small reduction in transmittance is caused by the greater energy dispersion of the 5 eV pass energy case, which causes some BSEs to strike the analyzer deflection plates.

Overall, the predicted performance of the optimized retarding field toroidal analyzer design, as tabulated in Table 1, is significantly better than the simulated characteristics of other high-energy resolution spectrometer designs. For instance, the best energy resolution simulated for the widely used retarding field hemispherical deflector analyzer (HDA) predicts it having an energy resolution of around 0.05% for an input angular spread of ±0.4° (in both the azimuthal and polar angular directions) [22]. The present retarding field toroidal energy analyzer is predicted to have several orders of magnitude higher transmittance for a comparable or better relative energy resolution performance, giving it a considerable signal-to-noise advantage.

**Figure 4:**
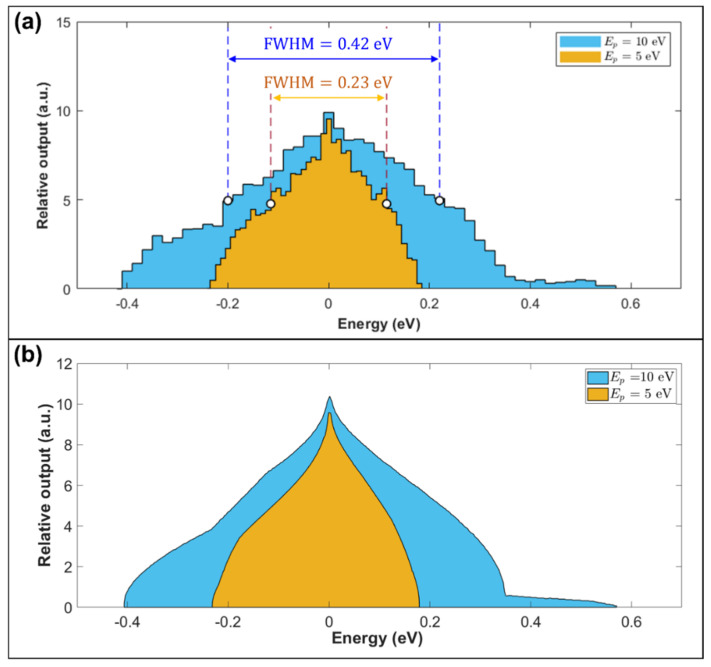
Simulated energy analyzer spectral distributions for the pass energies of 5 eV and 10 eV in the retarding field mode of operation. (**a**) Simulated histograms for the pass energies of 5 eV (bin size = 0.01 eV) and 10 eV (bin size = 0.02 eV). FWHM represents the full width half maximum value. (**b**) Simulated spectral distributions were obtained by an adaptive sampling method.

Figure 4a shows simulated spectral response distributions for the optimized retarding field toroidal energy analyzer design. They were obtained by plotting trajectories with a wide range of emission energies and polar angles around a 1 keV 45° central ray. Flat energy/polar angle distributions were assumed. A total of 10,000 trajectory rays were plot over an emission energy spread range of −0.45 eV to 0.6 eV in 0.0105 eV uniform steps, where the polar angular spread ranged from −6 to 6° in 0.12° steps for each emission energy. Two histograms were obtained, one for a pass energy of 10 eV, and the other for a pass energy of 5 eV. Their full width half maximum (FWHM) values of 0.42 eV at the 10 eV pass energy and 0.23 eV at the 5 eV pass energy approximately agree with their respective energy resolution values of 0.395 eV and 0.199 eV derived by the RMS spot size method. It is not clear which of these simulation estimates is more accurate. The FWHM estimates assume that the analyzer spectral response distribution can be represented by a Gaussian distribution, which is clearly not the case, particularly for the 5 eV pass energy. There is also the issue of which energy bin size to use in order to generate the spectral histograms shown in Figure 4a. The FWHM value for the 5 eV pass energy histogram changed from 0.27 eV for a bin size of 0.02 eV, to 0.23 eV for a bin size of 0.01 eV. These considerations would suggest that the RMS spot size method provides a better energy resolution estimate. In the present context, it is more important to note that these two different methods for estimating the energy resolution approximately agree.

The energy spectral distributions for the two pass energies were also computed via an alternative method that can more accurately determine the energy spectral distribution shape. The algorithm sweeps through a specified range of emission energies and, at each energy, calculates the detected polar angular interval. The intervals’ endpoints at each emission energy are in turn computed using a binary search method together with numerical ray-tracing. Using an emission energy range of 999.4 eV≤E≤1000.6 eV and a maximum of 9 bisection iterations per energy, a total of 9709 rays were used. The resulting spectral distributions in Figure 4b have a teardrop-like shape and are clearly non-Gaussian in form.

Finally, the analyzer design was evaluated for robustness against small perturbations of the electrodes’ geometry and voltages, which are important considerations for making the analyzer instrument in practice. For ±50 μm variations in the electrodes’ positions and thicknesses, the worst energy resolution obtained was 0.0405% (compared to the previous 0.0395% value), still well below the design’s 0.05% resolution target, while the pass energy shifted by a maximum of 0.18 eV. Additionally, when the plate voltages were varied by ±0.001%, corresponding to a 10^−5^ voltage ripple, the energy resolution worsened to 0.0399% while the pass energy changed by no more than 0.01 eV.

### 3.2. Toroidal Analyzer Performance in SE Mode

**Figure 5:**
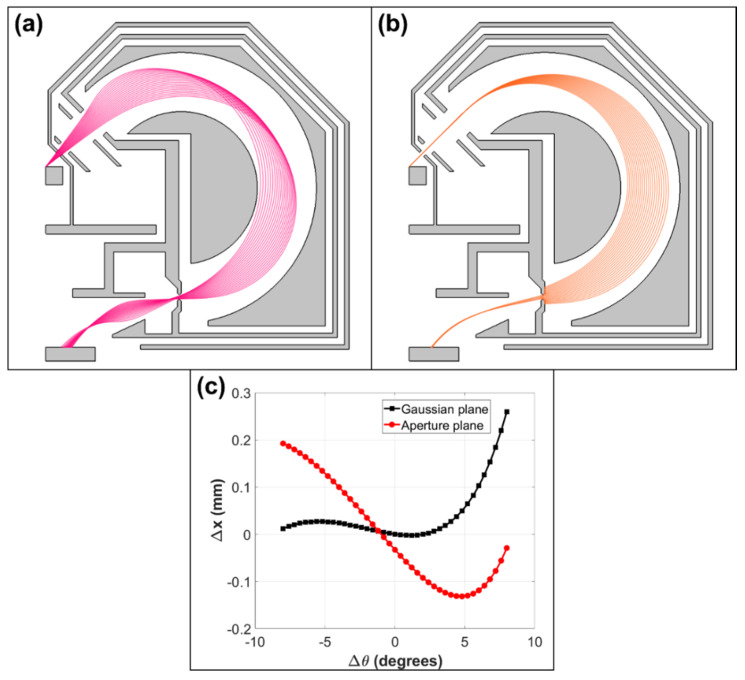
Simulation of the toroidal energy analyzer design operating in secondary electron acquisition mode. (**a**) SE trajectory paths emitted at 1 eV for the polar angular range of 37° to 53° in steps of 0.4°; (**b**) SE trajectory paths emitted for a polar emission angle of 45° for an emission energy from 0.9 to 1.1 eV in steps of 0.01 eV. (**c**) Exit distance (Δx) from the central ray as a function of the polar emission angular spread at the Gaussian plane and Aperture plane.

Since the present toroidal energy analyzer design was optimized to operate in a retarding field mode of operation, it will naturally no longer be optimal for the SEES application. Figure 5 presents the simulated trajectory ray paths at different emission energies and polar angles around a central SE ray of 1 eV and polar angle of 45°. The emitted energy ranges from 0.9 to 1.1 eV, and there is a polar angular spread of ±8°. While the energy dispersion characteristics are similar to the original analyzer design (Figure 5b), Figure 5a clearly indicates that the exit focal point lies beyond the exit aperture plane, which leads to the simulated spot size being slightly larger at the exit aperture plane than the Gaussian plane, as shown by Figure 5c. Table 2 provides more details, giving the electrode voltages as well the energy dispersion/resolution and focusing characteristics. It indicates that the simulated aberration limited energy resolution ($\Delta E_{AB}$) at the exit aperture plane is greater than its value on the Gaussian plane, 2.331% compared to 1.571%, respectively.

On the other hand, since the aperture width is relatively large (0.45 mm), the final simulated energy resolution of 5.334% is dominated by energy dispersion (4.799%), and not greatly compromised by the non-optimal focusing action.

**Figure 6:**
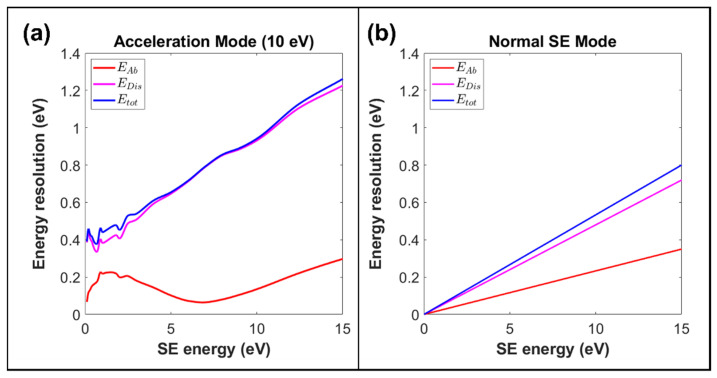
Simulated spectrometer energy resolution as a function of SE energy. Component aberration resolution and aperture energy dispersion parts of the total energy resolution are also shown. (**a**) Acceleration mode, where the pass energy of the SEs is increased by 10 eV. (**b**) Normal SE mode of operation, where the pass energy is equal to the SE energy.

Figure 6 presents the simulated energy resolution characteristics for a SE acceleration mode of operation, compared to the normal way of acquiring the SE energy spectral signal. The voltages $V_{D}$ and $V_{B}$ are adjusted to increase the SE pass energy by 10 eV. Since the energy dispersion at the exit aperture is proportional to the pass energy, the transmitted energy spread, $\Delta E_{DIS}$, will increase proportionally, causing the total detected energy spread, $\Delta E_{tot}$ of the low energy SEs (0 to 1 eV) to go up by more than an order of magnitude, as shown in Figure 6a,b. However, there will also be a corresponding gain in signal-to-noise performance. This gain in signal-to-noise performance has proved very useful for applications where only changes in the SE energy spectral shape need to be quantified [13,14,15]. The acceleration SE mode of operation in the present analyzer design can readily be implemented electronically by changing the values of $V_{B}$ and $V_{D}$. In the original SE toroidal energy analyzer design, the acceleration mode of operation was achieved by biasing the specimen negatively and surrounding the specimen with conical-shaped electrodes. The advantage of the present toroidal energy analyzer design is that it can be readily implemented without needing to bias the specimen and add extra electrodes.

Figure 6a,b indicate that the energy resolution of the redesigned analyzer, when applied to capturing the SE energy spectral signal is still aperture limited, both in the acceleration and non-acceleration modes of operation. Although the acceleration of SEs through the analyzer causes them to de-focus at the exit aperture plane, leading to an enlargement of the aberration limited energy resolution $\Delta E_{AB}$, the effect is largely suppressed since the total energy resolution is aperture limited.

## 4. Conclusions and Future Work

This paper has presented the design of a REELS attachment for LVSEM applications by carrying out a simulation of scattered electron trajectory ray paths. The spectrometer attachment is small enough to fit on to the specimen stage of an SEM and is designed to acquire nanoscale spatially resolved REELS information. For the capture of 1 keV BSEs emitted in the polar angular range between 40° to 50°, direct ray-tracing simulations predict that the spectrometer attachment will have an energy resolution of around 0.4 eV at a pass energy of 10 eV, and 0.2 eV at a pass energy of 5 eV. This predicted performance will make it a suitable REELS attachment for SEMs that use field emission electron sources. Simulations also predict that the same analyzer design can be applied to capture the SE energy spectrum, without any significant loss of performance.

How effectively REELS can be carried out in an SEM remains to be seen. The next step is to make a prototype of the analyzer attachment design and test it out experimentally.

Several implementation challenges remain, including the manufacture of the instrument to the required machine tolerances, sparkover mitigation, and the provision of using multiple high voltage supplies in the interior of a hermetically sealed SEM chamber. Simulation results suggest that these issues are not insurmountable, and that the tolerances needed for the design to meet its resolution and transmittance requirements are well within the capabilities of precision manufacturing processes and research-grade high voltage power supplies.

Aside from manufacturing issues, measures will also need to be taken to mitigate the effects of stray electric/magnetic fields, as is the case with all electron energy spectrometer designs. Like the previous SE energy analyzer, the new attachment’s physical prototype will include several sets of conducting cover plates to not only shield the analyzer’s interior from external electric fields, but also protect the SEM primary beam from the strong electric fields generated within the analyzer itself.

Experience with the previous analyzer also suggests that external magnetic fields will not significantly impact the new attachment’s operation. Due to its small size, the previous analyzer was not strongly affected by spatial variations in the geomagnetic field and was able to capture sub-eV SE energies reliably without the need for additional Mu-metal screening. Similar findings will likely apply to the new analyzer design since it has not changed dramatically in overall size or layout.

Finally, for REELS inside the SEM to be feasible, an effective strategy for reducing surface contamination at the specimen may also be needed. However, the energy analyzer design developed here is already a significant advance on its predecessor in that it opens up the possibility of analyzing higher scattered electron energies inside the SEM with greater precision. It may also find application as an add-on attachment in other related electron energy spectral instruments, such as Auger electron spectroscopy (AES) and X-ray photoelectron spectroscopy (XPS) systems, as well as Scanning Auger microscopes (SAMs).

## Figures and Tables

**Table 1:** Simulated energy resolution and energy dispersion characteristics of the optimized analyzer attachment design for 1 keV incoming BSEs retarded down to the pass energies of 10 eV and 5 eV. All focusing/dispersion parameters, unless otherwise stated, are evaluated on the exit aperture plane.

Parameter	$E_{p}=10\;\mathrm{eV}$	$E_{p}=5\;\mathrm{eV}$	Units
$V_{B}$	−990.000	−995.000	V
$V_{D}$	−996.565	−998.237	V
$V_{F1}$	−965.094	−959.238	V
$V_{F2}$	−846.882	−839.943	V
$V_{C1}$	−957.577	−1015.521	V
$V_{C2}$	−982.404	−992.471	V
$V_{MCP}$	0.0	0.0	V
Input polar angular spread, Δθ	±5.0	±4.7	°
Aberration spot size (2 × RMS)	0.309	0.303	mm
Energy dispersion D (Δx=DΔE)	1.382	2.727	$\mathrm{mm}\cdot {\mathrm{eV}}^{-1}$
$\Delta E_{AB}$ on the Gaussian Plane (2 × RMS)	0.0510	0.0199	%
$\Delta E_{AB}$(2 × RMS)	0.0224	0.0111	%
$\Delta E_{DIS}$(0.45 mm wide aperture)	0.0325	0.0165	%
$\mathrm{Total}\;\mathrm{exit}\;\mathrm{energy}\;\mathrm{resolution}\;\Delta E_{tot}$	0.0395	0.0199	%

**Table 2:** Simulated energy resolution and energy dispersion spectrometer characteristics of the analyzer attachment design in the secondary electron mode of operation.

Parameter	Value	Units
$V_{B}$	0.0	V
$V_{D}$	−( $0.688\times E_{p})$	V
$V_{F1}$	0.0	V
$V_{F2}$	0.0	V
$V_{C1}$	0.0	V
$V_{C2}$	0.0	V
$V_{MCP}$	100.0	V
Input polar angular spread, Δθ	±8.0	°
Spot size on aperture plane (2 × RMS)	0.217	mm
D	$9.378/E_{p}$	$\mathrm{mm}\cdot {\mathrm{eV}}^{-1}$
$\Delta E_{AB}$ on Gaussian plane (2 × RMS)	1.571	%
$\Delta E_{AB}$ on aperture plane (2 × RMS)	2.331	%
$\Delta E_{DIS}$ for a 0.45 mm wide aperture	4.799	%
$\mathrm{Total}\;\mathrm{energy}\;\mathrm{resolution}\;(\Delta E_{tot}$)	5.334	%
